# Gastroepiploic Vascularized Lymph Node Transfer for Extremity Lymphedema: Tips and Insights from Extensive Clinical Experience

**DOI:** 10.3390/medicina61030503

**Published:** 2025-03-15

**Authors:** Mirco Pozzi, Davide Di Seclì, Alberto Bolletta, Emanuele Cigna, Chiara Camilloni, Beniamino Brunetti, Paolo Persichetti, Michela Schettino, Luigi Losco, Hung-Chi Chen

**Affiliations:** 1 Plastic Surgery Unit, Department of Translational Research and New Technologies in Medicine and Surgery, University of Pisa, 56121 Pisa, Italy; alb.bolletta@gmail.com (A.B.); emanuele.cigna@gmail.com (E.C.); 2 Department of Plastic and Reconstructive Surgery, China Medical University Hospital, Taichung 404, Taiwan; davidedisecli@hotmail.it (D.D.S.); chiara.camilloni@unicampus.it (C.C.); michelaschettino@gmail.com (M.S.); luigi.losco@gmail.com (L.L.); 3 Surgery and Neuroscience-Plastic Surgery Unit, Department of Medicine, University of Siena, Policlinico Santa Maria “Le Scotte”, 53100 Siena, Italy; 4 Research Unit of Plastic, Reconstructive and Aesthetic Surgery, Department of Medicine and Surgery, Campus Bio-Medico University of Rome, 00128 Rome, Italy; b.brunetti@policlinicocampus.it (B.B.); p.persichetti@policlinicocampus.it (P.P.); 5 Unit of Plastic and Reconstructive Surgery, CHIREC de Braine L’Alleud Hospital, 1410 Brussels, Belgium; 6 Plastic Surgery Unit, Department of Medicine, Surgery and Dentistry, University of Salerno, 84081 Salerno, Italy

**Keywords:** lymphedema, gastroepiploic vascularized lymph node transfer (VLNT), lipectomy, SAL

## Abstract

*Background and Objectives*: Lymphedema is a chronic and progressive condition that leads to significant morbidity, including recurrent infections, fibrosis, and functional limitations. Conservative treatments often offer limited relief, particularly in severe cases. Vascularized lymph node transfer (VLNT), especially using the gastroepiploic lymph node flap, has emerged as a promising intervention. This study evaluates the long-term outcomes of gastroepiploic VLNT combined with suction-assisted lipectomy (SAL) for extremity lymphedema. *Materials and Methods*: A retrospective review was conducted on 53 patients treated for lymphedema at our clinic in Taiwan from January 2016 to August 2023. The inclusion criteria required patients to have persistent lymphedema for at least six months despite conservative treatment. VLNT was performed using a laparoscopic approach, and postoperative assessments included limb circumference measurements, lymphoscintigraphy, and tonicity evaluations. *Results*: Follow-up data were collected for a minimum of 12 months. At a mean follow-up of 14.2 months, significant reductions in limb circumference were observed—35.5% ± 24.9% for upper limbs and 32.2% ± 4.5% for lower limbs. Tonicity improved by 6.2%, and no cellulitis episodes were reported post-surgery. Minor complications included hematoma and sensory changes, with no major donor-site morbidity. Lymphoscintigraphy confirmed improved lymphatic drainage. *Conclusions*: Gastroepiploic VLNT combined with SAL is an effective and safe treatment for severe extremity lymphedema, providing significant improvements in limb size and tissue tonicity. This technique offers a promising solution for refractory cases.

## 1. Introduction

Lymphedema, whether primary or secondary, presents a significant therapeutic challenge due to its chronic nature, progressive worsening, and potential for debilitating complications such as recurrent infections, fibrosis, and functional limitations. Advances in the understanding of the lymphatic system’s anatomy and pathophysiology, coupled with innovations in surgical techniques, have led to more effective treatments aimed at alleviating the symptoms and improving the quality of life of patients. Among these, vascularized lymph node transfer (VLNT) has gained prominence as a powerful intervention for severe cases where conservative treatments are insufficient [1,2,3,4].

VLNT involves the transplantation of functional lymph nodes to an affected extremity, with the nodes’ vascular supply maintained via microanastomosis, thereby restoring physiological lymphatic drainage [2,3,4,5]. This technique has been shown to promote lymphangiogenesis, the creation of new lymphatic pathways, and enhance lymphatic-venous drainage through VEGF-C secretion. Historically, the deep inferior epigastric artery perforator (DIEP) and superficial inguinal lymph node flaps have been the most common donor sites, particularly for postmastectomy lymphedema. However, other sites, including the gastroepiploic lymph nodes, have emerged as valuable alternatives, especially in cases involving lower extremity lymphedema [6,7,8].

The gastroepiploic lymph node flap, harvested laparoscopically from the omentum, has proven to be an advantageous option due to its reduced donor-site morbidity and versatility [3]. It offers a minimally invasive approach that significantly lowers the risk of iatrogenic lymphedema in the upper or lower limbs, a potential complication when harvesting from superficial lymph node basins [9,10,11,12,13,14]. The gastroepiploic lymph node flap, with an average size of 3 × 7 cm, allows for placement in the distal areas of the extremity, maximizing the gravitational advantage in enhancing lymphatic drainage. Furthermore, studies have shown that the use of gastroepiploic lymph nodes leads to significant improvements in limb circumference, symptom relief, and patient satisfaction, with reductions in lymphedematous limb volume reaching up to 43.7% at follow-up [2,3].

Our extensive clinical experience with the gastroepiploic VLNT, particularly when combined with suction-assisted lipectomy (SAL), has yielded remarkable long-term results. This approach not only helps to re-establish lymphatic drainage but also addresses the adipose tissue hypertrophy that frequently accompanies chronic lymphedema. Moreover, innovations in laparoscopic and robotic-assisted harvest techniques have further reduced complications such as postoperative pain, gastrointestinal issues, and donor-site morbidity [15,16]

In this article, we present a detailed overview of our clinical approach to managing extremity lymphedema using gastroepiploic vascularized lymph node transfer. Drawing from extensive clinical cases, we aim to offer practical tips, technical insights, and long-term outcome data to optimize the use of this procedure in lymphedema management. By doing so, we hope to enhance the efficacy and safety of VLNT and provide a valuable resource for surgeons seeking to refine their approach to treating this complex condition.

## 2. Materials and Methods

A retrospective chart review of patients was performed for individuals treated for lymphedema at our clinic in Taiwan between January 2016 and August 2023. Each patient underwent a clinical evaluation. The inclusion criteria focused on patients with lymphedema that had persisted despite conservative treatment for at least six months. The exclusion criteria applied to those with a history of vascular malformations, prior surgeries on the affected limbs, trauma, previous lymphaticovenous anastomosis (LVA) procedures, or candidacy for LVA. Additionally, the exclusion of the upper extremities from the study was performed to avoid potential bias, as the focus was on the lower extremities only. Finally, the patients were excluded if their demographic or clinical data (such as age, sex, smoking status, medication use, surgical indications, and procedures) or follow-up information was incomplete or unavailable.

Before surgery, all the patients had their limb circumferences, body weight, and tonicity assessed. The circumferences of the lower limbs were measured at specific levels: 10 cm above the knee (AK), 10 cm below the knee (BK), 10 cm above the ankle (AA), and at the midfoot (F). These measurements, along with serial photographs, were utilized for an objective clinical evaluation.

Preoperative and 6–8-month postoperative lymphoscintigraphy was conducted to assess lymphatic function. Additionally, a computed tomography (CT) scan of the limb was performed three months after surgery to verify the viability of the flaps.

Tonicity was measured using a tonometer with a 10 mm diameter central plunger, applying a weight of 30 g (0.38 g/mm^2^, 0.37 cN/mm^2^), at two locations on each limb. Preoperative lymphoscintigraphy was conducted to evaluate lymphatic drainage in the lower limbs. The frequency of cellulitis episodes was documented before and after the procedure.

Follow-up assessments were conducted at 1 month, 6 months, 8 months, and 1 year post-surgery, with a minimum follow-up duration of 1 year.

The study received approval from the Research Ethics Committee (approval number CMUH110-REC1-004), and the principles of the Helsinki Declaration were strictly followed. Written informed consent for data analysis and publication was obtained from all the participants.

All the cases included in this study were performed at the China Medical University Hospital in Taiwan, with Professor Chen acting as the lead surgeon. The other surgeons who assisted in these procedures were trained in Taiwan.

Statistical analysis was performed with the SPSS software v. 22 (IBM Corp., Armonk, NY, USA).

### Surgical Technique

**Figure 1:**
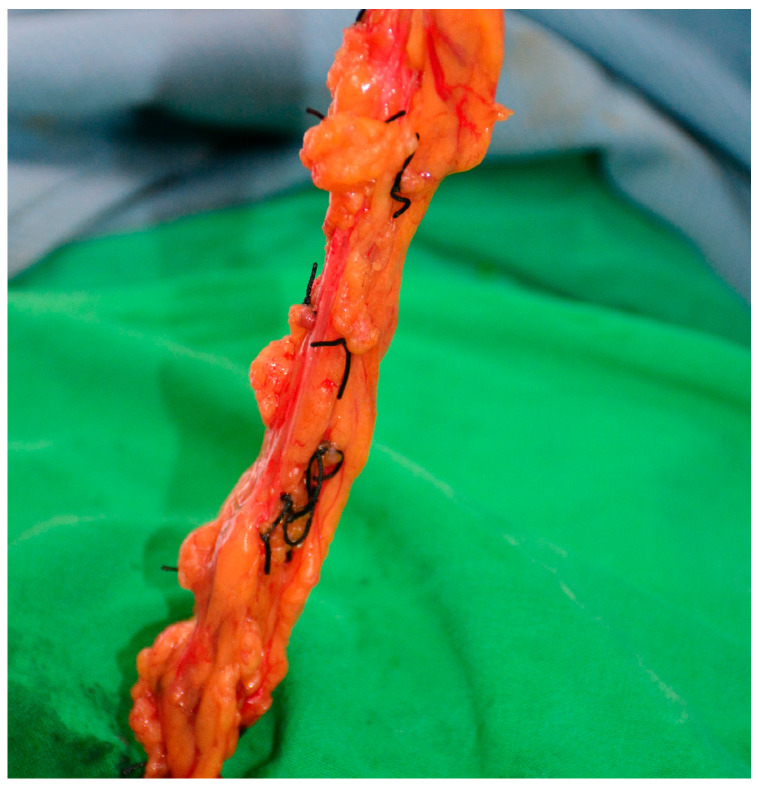
View of the gastroepiploic flap in its full length, highlighting the tissue’s extent.

**Figure 2:**
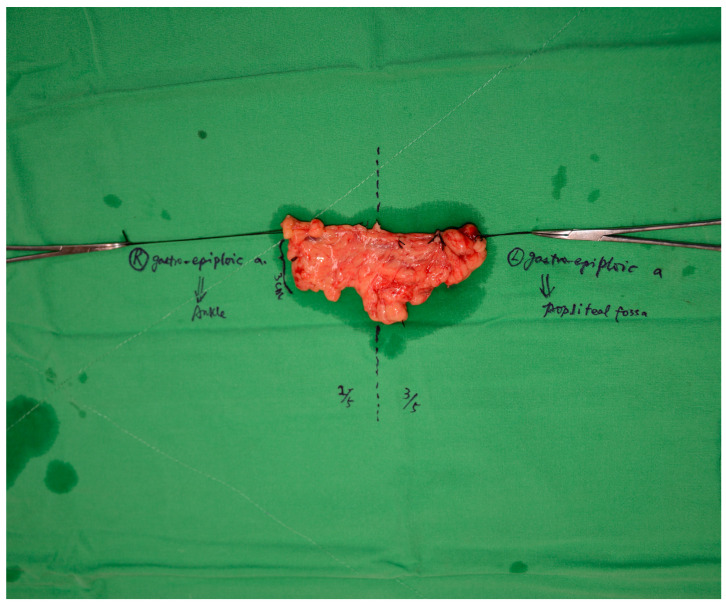
The gastroepiploic flap prior to being divided into two. The asymmetrical division is visible, along with the respective placement sites in the limb for unilateral lymphedema treatment.

For the harvest of the right gastroepiploic vascularized lymph node (VLN) flap, the patients were positioned according to the affected limb: supine for lower limb lymphedema and with arms at 90° angles for upper limb lymphedema. The pneumoperitoneum was established using a supraumbilical 12 mm trocar, followed by the placement of two 5 mm trocars in the right and left upper quadrants. Reverse Trendelenburg positioning aids in visualizing the omentum and the right gastroepiploic vessels. Under the guidance of the plastic surgery team, an experienced laparoscopic surgeon performed meticulous dissection using a 5 mm LigaSureDolphin Tip Laparoscopic dissector. The right gastroepiploic vessels were carefully identified, and the omentum was freed from the stomach and transverse colon to preserve the surrounding lymphatic and vascular integrity. The harvested flap, approximately 15 cm long and 3–4 cm wide, was retrieved through an enlarged umbilical port and prepared under a microscope. It was divided into two different-sized flaps, with the vascular and lymphatic structures precisely ligated. See Figure 1 and Figure 2.

**Figure 3:**
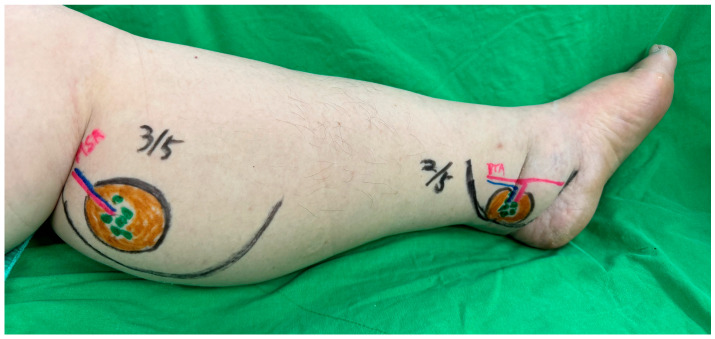
The preoperative marking outlines the planned surgical sites for anastomoses in the cases of unilateral lymphedema.

**Figure 4:**
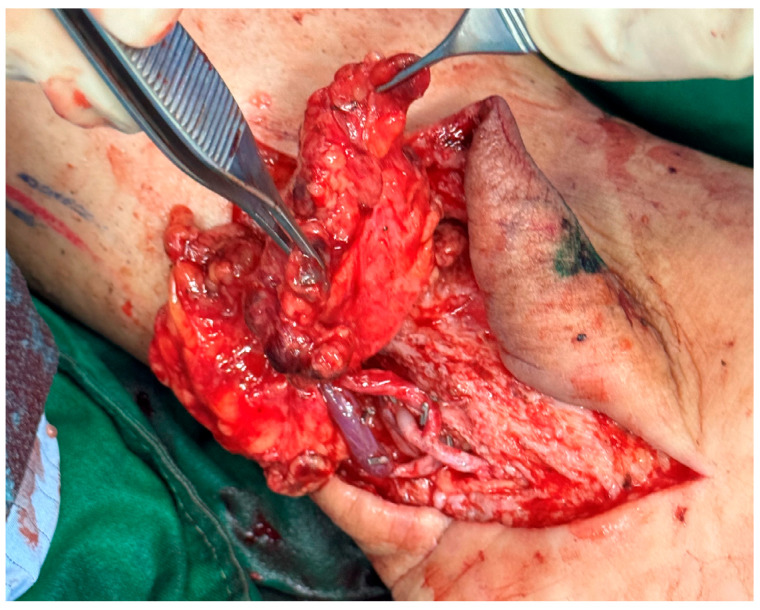
The intraoperative picture displays the gastroepiploic vascularized lymph node flap anastomosed at the ankle level in unilateral lymphedema.

Microsurgical anastomoses were performed using the posterior tibial and medial sural vessels for lower limbs, with split-thickness skin grafts applied to avoid compression over the inset flaps. See Figure 3 and Figure 4.

Postoperative monitoring included clinical evaluation, Doppler ultrasound, and observation for two days in an intensive care unit, followed by discharge within a week. Two weeks after the first surgery a suction-assisted lipectomy was performed.

## 3. Results

Between January 2016 and August 2023, a total of 53 patients with lymphedema were treated in our hospitals in Taiwan. The average age of the patients was 48.4 years (range: 32–68 years), with a female–male ratio of 47% to 6%. Smoking status was recorded for five patients (11.9%), and medication use was documented for seven patients (16.6%). Among these, 30 individuals (47.6%) presented with unilateral lymphedema, while 23 (52.4%) had bilateral disease. A total of 37 patients were classified as stage II (69.8%), 12 as stage III (22.4%), and 4 as stage IV (7.5%) lymphedema. In total, 30 individuals (56.60%) had post-cancer lymphedema, 2 (3.77%) had post-thigh lift lymphedema, 15 (28.30%) had iatrogenic lymphedema, and 6 (11.32%) had primary lymphedema (See Table 1).

In the unilateral group, all the patients underwent vascularized lymph node transfer (VLNT), with supplementary suction-assisted lipectomy (SAL). The flap was divided into two distinct halves to optimize its use. The proximal half, which contains a higher density of lymph nodes, was always positioned at the ankle to maximize lymphatic function in this critical area, while the distal half was consistently placed at the level of the knee. Vascular anastomoses were carefully performed to ensure reliable perfusion of the flap. In the popliteal fossa, the medial sural artery and vein were connected to the flap vessels in an end-to-end configuration. At the ankle, the vascular connections were established using an end-to-side technique with the posterior tibial artery and vein, ensuring adequate perfusion. This approach allowed for the precise placement of the lymph node-rich segment of the flap at the ankle in all cases while ensuring the distal segment was effectively positioned at the knee.

In the bilateral group, the patients received bilateral procedures. The gastroepiploic flap was divided into two portions and anastomosed at the ankle level for each limb. The proximal portion of the flap, which contains a higher concentration of lymph nodes, was strategically placed in the limb more severely affected by lymphedema to maximize therapeutic benefit. Among these cases, the more affected limb was the right one in 58% of the patients and the left one in 42%. This tailored approach ensured the optimal placement of the lymph node-rich portion to address the greater lymphatic demand. All the patients of the bilateral group underwent SAL after two weeks.

All the patients were followed for a minimum of 12 months, with an average follow-up duration of 14.2 months (range: 12–52 months). The mean circumference reduction rate was 35.5% ± 24.9% for the upper limb and 32.2% ± 4.5% for the lower limb. The limb circumference reductions after VLNT and SAL averaged 4.9 cm (range: 3.1–7 cm). Specific reductions were observed as follows:

- Mid-thigh: A total of 5.9 cm (range: 4.8–7 cm) in unilateral and 5.2 (range 4.1–6.9 cm) in bilateral case.
- Mid-calf: A total of 4.6 cm (range: 2.8–6.7 cm) in unilateral and 4.3 (range 2.6–6.8 cm) in bilateral case.
- Ankle: A total of 3.9 cm (range: 2.7–4.4 cm) in unilateral and 3.7 (range 2.8–4.3) in bilateral case.
- Mid-foot: A total of 1.4 cm (range: 0.7–2.1 cm) in unilateral and 1.3 (range 0.6–2.2 cm) in bilateral case.

Tissue tonicity improved by an average of 6.2% (range: 5.4–7.2%) in the unilateral and 6.3% (range 5.2–7.9%) in the bilateral cases, reflecting a notable softening of the tissues as reported by both the physicians and patients.

Even though a direct comparison between the bilateral and unilateral cases is quite difficult, the circumference reduction achieved at the distal aspect of the extremity is quite similar. However, the improved circumference reduction at the proximal half of the limb with a double inset versus the single inset should suggest a possible advantage of the double VLN transfers.

Before treatment, the patients experienced an average of three episodes of cellulitis annually (range: 1–5). Following treatment, no episodes of cellulitis were observed in the treated limbs during the follow-up period.

The average time required for flap harvesting was 42.4 min, while the mean total surgical time was 184 min (range: 150–235 min). The flap pedicle length averaged 7.35 cm (range: 6–8 cm), and the mean flap size was 33.2 cm^2^ (range: 22–42 cm^2^). Skin graft dimensions averaged 3 × 8 cm^2^.

There were no major complications except for one failure. No patients exhibited complications such as gastric outlet obstruction, gastroparesis, gastric dysmotility, or colonic ischemia following the transfer of the right gastroepiploic lymph node flap, and all resumed their diet on the first postoperative day. Minor complications were local hematoma, numbness, hyperesthesia, and unsightly scar which were treated conservatively at first with local injection of steroid/Botox. In the lower limb, five cases had debulking procedures and scar revision.

Postoperative lymphoscintigraphy showed improved lymphatic drainage in all the cases.

A follow-up abdominal CT scan conducted one year postoperatively revealed no donor-site morbidity in any of the patients. Seven cases of partial skin graft loss required re-grafting during the postoperative period. 

**Figure 5:**
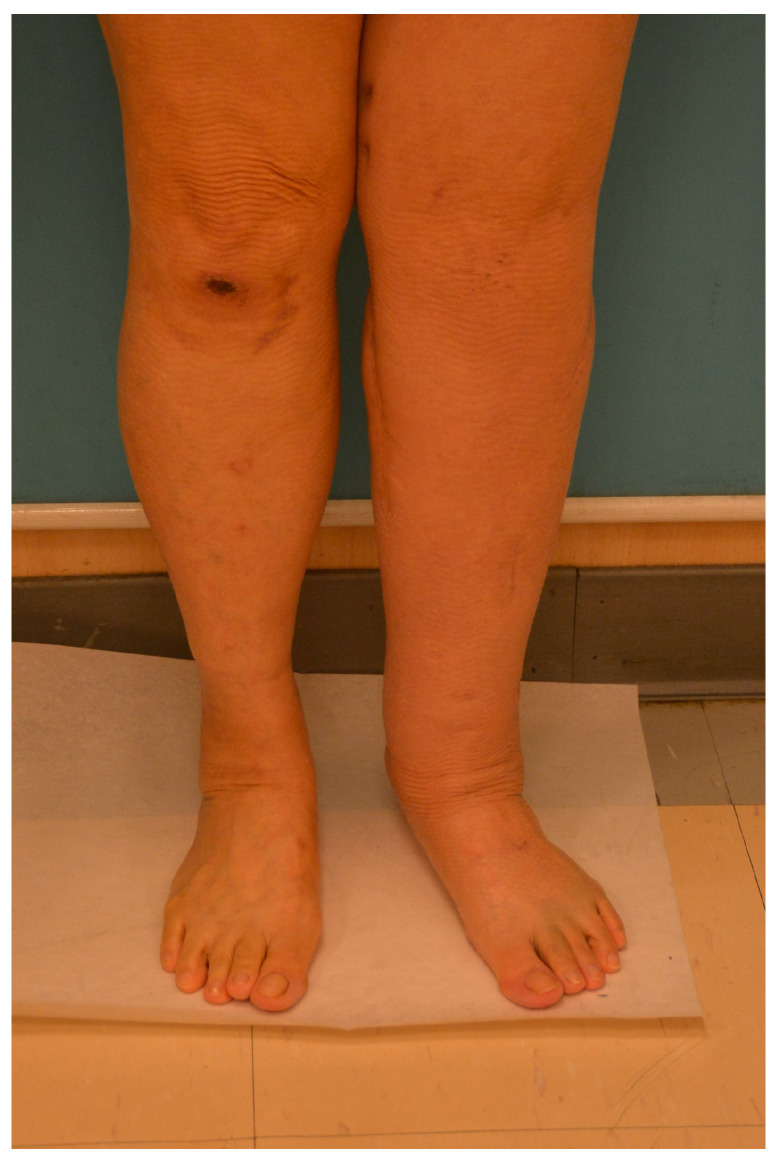
The 56-year-old woman was operated for cervical cancer 5 years ago. She developed lymphedema of the left lower limb 3 years later. This was the anterior view. The circumference measurements of bilateral lower limbs were 49.5 (right)/56 (left) cm at the level of mid-thigh; 37.5 (right)/40.5 (left) cm at the level of mid-leg; 21 (right)/22.5 (left) cm at the level of the ankle; and 22 (right)/22.5 (left) cm at the level of the foot.

**Figure 6:**
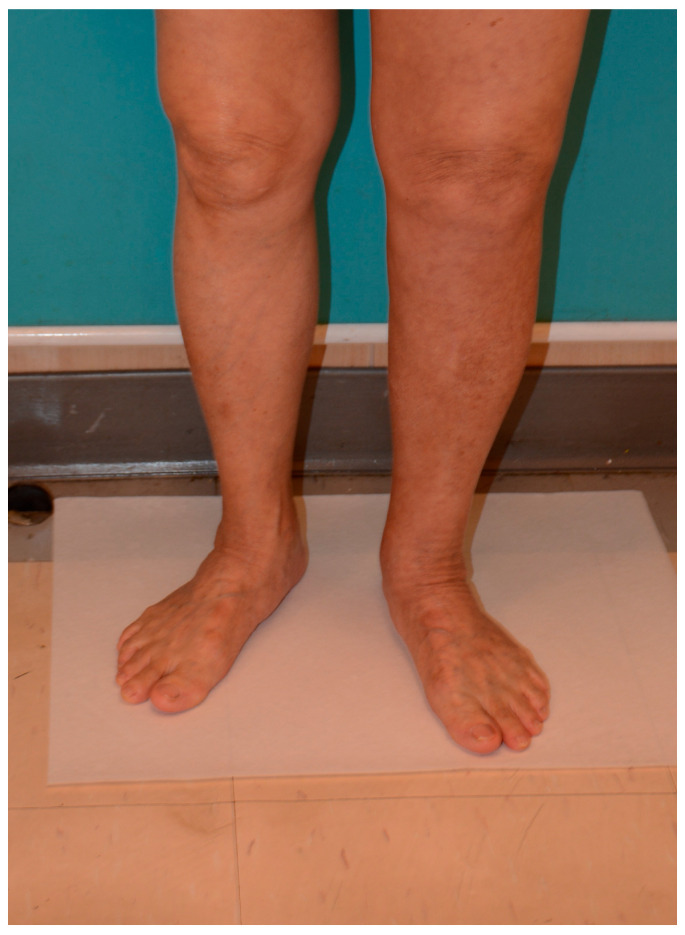
One year after surgery, the discrepancy between 2 lower limbs was 2.6 cm at the level of mid-thigh; 2.1 cm at the level of mid-leg; 1.8 cm at the level of the ankle, and 1.2 cm at the level of the foot.

The relationships between the demographic characteristics of the patients and the rates of complications were statistically analyzed; however, no statistically significant associations were found. The statistical significance was defined as *p* < 0.05 (See Figure 5 and Figure 6.)

## 4. Discussion

At our center, laparoscopic gastroepiploic lymph node flap harvesting has become the preferred method because of its lower operative morbidity, faster recovery time, and reduced risk of complications such as postoperative pain, gastrointestinal dysfunction, and donor-site morbidity. Laparoscopy, while technically challenging, offers several advantages over traditional open methods. The small incision, typically made in the supraumbilical region, provides optimal access to the right gastroepiploic vessels without the need for larger incisions, minimizing the risk of infection and hernia. In addition, the pneumoperitoneum allows better visualization of the flap, facilitating precise dissection and preservation of vessels and lymphatic tissue [17]. This technique is suitable for patients without extensive adhesions from previous abdominal surgery or trauma. However, if there is significant scarring or previous surgery, a mini-laparotomy may be considered [18]. This alternative approach is equally effective in flap harvesting, although it involves slightly higher risks and longer recovery time than the laparoscopic method. The possibility of the gastroepiploic flap being divided into multiple components offers great flexibility in meeting the specific needs of each patient. In our series, we have primarily divided the flap into two portions: a lymph node-rich proximal segment and a larger distal segment, with the proximal 2/5 being denser in lymph nodes and the distal 3/5 containing fewer lymph nodes but greater volume. This asymmetric division allows for the optimized placement of lymph nodes according to the patient’s needs. This division optimizes the function of the flap by ensuring that the lymph node-dense portion is strategically placed in areas that require the most lymphatic drainage, such as the distal lower limb (ankle and knee). The more distal portion, which is usually larger and less lymph node dense, is placed more proximally to accommodate the greater volume of tissue required in these areas. The choice of placement site is critical to maximize the functional benefits of the procedure [4,19,20]. In our experience, dual-level VLNT, which involves placing one portion of the flap at the ankle and the other at the knee, has provided excellent results in cases of both unilateral and bilateral lymphedema. This approach takes advantage of the gravitational pull on lymphatic fluid, ensuring that the most effective portion of the flap is located in the most distal area of the limb, where lymphatic drainage is most compromised. By positioning the proximal, lymph node-rich segment at the ankle and the distal segment at the knee, we can achieve significant improvement in both the upper and lower extremity lymphedema, optimizing the effectiveness of the transfer. For vascular anastomosis, the posterior tibial artery and medial sural vessels are preferred in the lower limb. The end-to-side anastomosis at the ankle level ensures that the flap receives adequate perfusion, while the end-to-end anastomosis at the knee level ensures optimal flow to the larger, less lymph node-dense part of the flap. These anastomoses have demonstrated durability in our experience, contributing to the long-term success of the procedure.

Our study demonstrates significant improvements in limb circumference and tissue tone following gastroepiploic VLNT combined with suction-assisted lipectomy (SAL). The mean reduction in limb circumference was 35.5% ± 24.9% for upper limbs and 32.2% ± 4.5% for lower limbs. These reductions are in line with, or even greater than, the results reported in other studies, highlighting the effectiveness of this technique in the management of chronic and severe lymphedema. Importantly, the improvement in tissue tone, which was observed as an increase of 6.2 percent in the unilateral group and 6.3 percent in the bilateral group, reflects the softening of fibrotic tissue that often accompanies long-standing lymphedema. The complete resolution of cellulitis episodes in the treated limbs in the postoperative period is a particularly striking result, as recurrent cellulitis is a common and debilitating complication of lymphedema. Restoration of lymphatic drainage through functional lymph node transplantation likely contributed to the reduction in the incidence of these infections, which previously occurred with an average frequency of 3 episodes per year. This finding underscores the importance of VLNT in addressing not only the cosmetic and functional aspects of lymphedema, but also the prevention of serious complications such as infection and fibrosis.

In terms of complications, the gastroepiploic VLNT procedure is generally well tolerated, with no major complications observed in our cohort. The most common minor complications included local hematomas, numbness, and hyperesthesia at the donor and recipient sites. These were managed conservatively with steroid injections and botox therapy. Importantly, no cases of gastric outlet obstruction, gastroparesis, or other gastrointestinal complications, such as colonic ischemia, were observed in any patient. This reinforces the safety of laparoscopic retrieval from the omentum, which preserves the integrity of the gastrointestinal system. However, partial loss of the skin graft occurred in some patients, requiring a new graft, although this complication was minor and easily resolved. Notably, donor-site morbidity was minimal, with no significant complications in the abdomen at 1-year follow-up, as confirmed by abdominal CT. This is an important consideration, as reduced donor site morbidity is a critical advantage of the gastroepiploic flap over other donor sites, such as the DIEP flap or inguinal lymph nodes. Despite the generally favorable results, there are areas where technical refinements could further improve the effectiveness of the procedure. For example, improvements in flap harvesting techniques through advanced robot-assisted methods could provide even greater precision and reduce surgery time [20,21,22,23].

In addition, preoperative lymphoscintigraphy and CT can be used to better assess lymph flow and flap viability, potentially guiding the surgeon toward more personalized approaches to flap design and placement. The use of gastroepiploic VLNT for severe extremity lymphedema represents a significant step forward in the treatment of this difficult condition. Our experience with laparoscopic flap harvesting combined with suction-assisted lipectomy has yielded excellent clinical results in terms of limb circumference reduction, improved tissue tone, and the prevention of infection. The ability to divide the flap and tailor its positioning to the patient’s specific anatomical needs is critical to the success of the procedure. The technique’s versatility, coupled with low donor-site morbidity, makes it an ideal option for managing severe lymphedema in patients unresponsive to conservative therapies.

In addition to our own findings, it is important to position our results within the broader context of existing research on lymphedema treatment. Lymphovenous anastomosis (LVA) has emerged as a widely studied surgical option for managing lymphedema, with several studies demonstrating its effectiveness in improving lymphatic drainage and reducing limb volume. While LVA has shown promising results, its success largely depends on patient selection, the extent of lymphatic damage, and the timing of the intervention. Other approaches, such as microsurgical lymphatic drainage and advanced compression therapies, have also shown varying levels of success in improving lymphatic function and reducing limb volume [1,24,25,26,27,28,29,30].

While our study provides valuable insights into gastroepiploic vascularized lymph node transfer for extremity lymphedema, its retrospective nature and lack of a control group introduce potential biases that may limit the generalizability of the findings. The single-center design further restricts the evidence’s robustness, highlighting the need for prospective, multi-center, randomized controlled trials. Additionally, the use of robotic surgery for lymph node harvesting could improve the precision and efficiency of the procedure, representing a promising direction for future research to refine the technique and strengthen its place in lymphedema treatment.

## 5. Conclusions

In conclusion, gastroepiploic vascularized lymph node transfer (VLNT) offers a highly effective treatment for refractory lymphedema, especially in cases involving the lower extremities. The ability to divide the flap into asymmetrical portions for double-level placement optimizes lymphatic drainage and addresses both functional and aesthetic concerns. Our results demonstrate significant improvements in limb circumference, tissue tonicity, and reduced cellulitis episodes. Laparoscopic harvesting minimizes donor-site morbidity, making this technique both safe and efficient. Overall, gastroepiploic VLNT represents a valuable addition to the armamentarium for managing severe lymphedema.

## Figures and Tables

**Table 1:** Patient’s demographics.

Demographics	
N. patients	53
Median Age	48.4 (32–68)
Smoke	5 (11.9%)
Medication	7 (16.6%)
Unilateral Lymphedema	30 (56.6%)
Bilateral Lymphedema	23 (43.4%)
Stage II	37 (69.8%)
Stage III	12 (22.6%)
Stage IV	4 (7.5%)
Cellulitis	3 (1–5)

## Data Availability

The data presented in this study are available on request from the corresponding author.

## Abbreviations

The following abbreviations are used in this manuscript:

VLNT	vascularized lymph node transfer
SAL	suction-assisted lipectomy
